# Assessment of Eating Disorders and Self-Esteem in Adolescents

**DOI:** 10.1192/j.eurpsy.2025.1153

**Published:** 2025-08-26

**Authors:** Z. Nesrine, D. Ben Touhemie, J. Boudabous, I. Hadjkacem, K. Chiha, H. Ayédi, K. Khemekhem, Y. Moalla

**Affiliations:** pédopsychitrie, Hedi chaker hospital, Sfax, Tunisia

## Abstract

**Introduction:**

Self-esteem is a significant indicator of health and well-being, as well as an explanatory variable for various aspects of human behavior. The relationship between self-perception, self-esteem, overall mental well-being, and eating disorders (ED) is considered particularly important, especially during adolescence.

**Objectives:**

to evaluate the relationsheep between eating disorders (ED) and self-esteem among adolescents and identify associated factors.

**Methods:**

This was a cross-sectional, descriptive, and analytical study conducted between June 2024 and September 2024 among adolescents resident in Sfax. Data were collected through an online Google Forms questionnaire exploring sociodemographic and relational data. We used the “Eating Attitudes Test 40 (EAT-40)” to detect the presence of ED, with a score of ≥ 30 indicating the presence of an eating disorder. Self-esteem was assessed using the Rosenberg Self-Esteem Scale: A score below 25 indicates very low self-esteem. A score between 25 and 30 indicates low self-esteem. A score between 31 and 33 indicates average self-esteem. A score between 34 and 39 indicates high self-esteem. score above 39 indicates very high self-esteem.

**Results:**

We collected data from 120 adolescents, with an average age of 16 years and a predominance of females (71.7%). The majority of participants (79.3%) lived in urban areas, 69.2% had a medium socioeconomic status, and 80.8% were enrolled in secondary schools, 33.3% of adolescent have reported relational difficulties with peers,41,5% of Adolescents were living in particular family situations( death of one parent, parental separation, relational difficulties with parents). In our study, 30% of adolescents had personal psychiatric histories. The prevalence of eating disorders estimated at 47.5%. Among participants, 43.3% had very low self-esteem, 55.8% had low self-esteem, and 0.8% had average self-esteem. Our study objectified a signifiant correlation between eating disorders and low self-esteem (p=0.005).

**Conclusions:**

The results of the study show a high prevalence of eating disorders among adolescents, associated with several factors, notably low self-esteem. This underscores the need to develop prevention strategies focused on improving self-esteem during adolescence. Developing effective interventions in this regard could be beneficial for addressing the behaviors and attitudes observed in eating disorders.

**Disclosure of Interest:**

None Declared

